# Engineered Tolerance: Tailoring Development, Function, and Antigen-Specificity of Regulatory T Cells

**DOI:** 10.3389/fimmu.2017.01460

**Published:** 2017-11-03

**Authors:** Nicholas A. J. Dawson, Jens Vent-Schmidt, Megan K. Levings

**Affiliations:** ^1^Department of Medicine, University of British Columbia, Vancouver, BC, Canada; ^2^BC Children’s Hospital Research Institute, Vancouver, BC, Canada; ^3^Department of Surgery, University of British Columbia, Vancouver, BC, Canada

**Keywords:** regulatory T cells, chimeric antigen receptors, T cell receptor, IL-2, autoimmunity, transplantation, inflammatory bowel disease, immunotherapy

## Abstract

Regulatory T cells (Tregs) are potent suppressors of immune responses and are currently being clinically tested for their potential to stop or control undesired immune responses in autoimmunity, hematopoietic stem cell transplantation, and solid organ transplantation. Current clinical approaches aim to boost Tregs *in vivo* either by using Treg-promoting small molecules/proteins and/or by adoptive transfer of expanded Tregs. However, the applicability of Treg-based immunotherapies continues to be hindered by technical limitations related to cell isolation and expansion of a pure, well-characterized, and targeted Treg product. Efforts to overcome these limitations and improve Treg-directed therapies are now under intense investigation in animal models and pre-clinical studies. Here, we review cell and protein engineering-based approaches that aim to target different aspects of Treg biology including modulation of IL-2 signaling or FOXP3 expression, and targeted antigen-specificity using transgenic T cell receptors or chimeric antigen receptors. With the world-wide interest in engineered T cell therapy, these exciting new approaches have the potential to be rapidly implemented and developed into therapies that can effectively fine-tune immune tolerance.

## Introduction

Regulatory T cells (Tregs) are essential to maintain self-tolerance and dampen immune responses during infection (1, 2). The best characterized subset of Tregs is defined by high expression of CD25 and FOXP3, the master-regulator of their phenotype and suppressive function (3). The critical role of FOXP3 in controlling Treg development and function is illustrated by the study of Tregs from patients with immunodysregulation polyendocrinopathy enteropathy X-linked (IPEX) syndrome (4). Depending on the specific mutation, IPEX patients may or may not have circulating FOXP3^+^ T cells, but even if FOXP3^+^ T cells are present, they are functionally defective due to inadequate FOXP3 transcriptional function (5–7).

Mechanistically, Tregs suppress the proliferation and function of many immune cells, even at very low Treg:effector cell ratios (2). In terms of suppressive pathways, multiple possibilities have been described, such as immunosuppressive cytokines, contact-dependent cytotoxicity, metabolic disruption, and suppression of antigen presenting cells *via* co-inhibitory molecule expression. Focusing on human Tregs, there is a dominant role for CTLA-4 and TGF-β. Monogenic mutations affecting CTLA-4 or proteins in its pathway affect Treg function (8, 9) and antibodies that block activation of TGF-β by human Tregs prevent their ability to control xenogeneic graft-versus-host disease (GVHD) (10). An additional aspect of Treg mechanisms is their ability to take on characteristics of other T helper (Th) cells (11, 12) resulting in sub-specialization and enhanced suppression of the Th cell subset they mirror (13). Whether or not these sub-specialized Tregs have unique suppressive mechanisms or are simply better able to traffic to the relevant sites of inflammation remains to be defined.

The immunosuppressive properties of Tregs make them attractive candidates for cellular therapy, particularly for application in conditions such as hematopoietic stem cell transplantation (HSCT), solid organ transplantation, and autoimmunity. However, harnessing Tregs for this purpose has not been trivial due to limitations related to cell isolation and expansion. In this review, we summarize the current state of Treg therapy in the clinic and discuss how engineering strategies can be used to improve upon current approaches.

## Current Treg Clinical Trials

There are two main approaches to increase Treg numbers and function: *in vivo* “boosting” using small molecules or proteins and adoptive cellular therapy. To date, the most successful strategy to “boost” Treg *in vivo* is the use of low-doses of IL-2. When given in limiting concentrations, IL-2 preferentially expands CD25^hi^ Tregs without significantly affecting cells expressing low-levels of CD25, such as resting conventional T (Tconv) cells or NK cells. This concept was first tested for treatment of hepatitis-C-virus-induced vasculitis where low doses of IL-2 induced an increase in circulating Tregs and clinical improvements in 8 of 10 patients (14). Subsequently, the beneficial effect of low-dose IL-2 therapy was also observed in GVHD, alopecia areata, type 1 diabetes (T1D), and systemic lupus erythematosus (15–19). However, a cautionary note is that in one study of T1D where IL-2 therapy was combined with rapamycin, there was an unexpected expansion of NK cells and worsening of disease (20). Thus, this approach may need further refinement to reduce the risk of expanding non-Tregs. Low-dose IL-2 and other strategies for *in vivo*-boosting of Tregs are discussed extensively in Zhang et al. and Boyman et al. (21, 22).

An alternate to *in vivo*-boosting is adoptive therapy with *ex vivo*-enriched, often expanded, Tregs. This method aims to overcome defective or low numbers of Tregs by transfer of a large number of Tregs to re-set the Treg:Tconv cell balance. Adoptive Treg therapy has been applied in the clinic for many years. The first successful study reported that chronic GVHD patients treated with Tregs had a significant reduction in clinical symptoms and immunosuppression (23). Subsequently, Treg therapy has been tested in several other GVHD cohorts, overall showing that infusion of autologous or third party (partially HLA-matched) Tregs is well tolerated, does not inhibit graft-versus-leukemia, and may be protective from GVHD (24, 25).

Adoptive transfer of Tregs has also been applied successfully in autoimmunity and organ transplantation. Children with T1D who received Tregs showed slowed disease progression and long-term preservation of residual beta-cells (26, 27). Adoptive transfer of Tregs in adults with T1D is also well tolerated, with evidence that the cells persist long term (>1 year) (28). A clinical trial of *in vitro*-expanded naïve Tregs is also underway in Crohn’s Disease, the first application of FOXP3^+^ Treg immunotherapy for inflammatory bowel disease (IBD) (ISRCTN97547683) (29). In addition, several clinical trials are testing autologous polyclonal or antigen-expanded expanded Tregs in kidney or liver transplantation; these trials are reviewed extensively in Ref. (30–33). To date, all of these studies have shown that adoptive Treg therapy in humans is feasible and safe, and initial data suggest that this approach may also be effective.

## Engineering IL-2

With the early success of low-dose IL-2 therapy as an approach to expand Tregs *in vivo*, there are now several efforts to improve upon this approach by modulating the way IL-2 interacts with its receptors. One strategy to modulate IL-2 is to use IL-2/anti-IL-2 monoclonal antibody (mAb) combination therapy to form “IL-2 complexes” that enhance the half-life of IL-2 after intravenous injection and provide preferential selection of certain immune cell subsets. For example, IL-2 in complex with anti-IL-2 mAbs, JES6-1A12 (mouse), or 5344 (human), preferentially expands Tregs, but not other IL-2-dependent cells such as CD8^+^ T and NK cells (34). This approach enriches Tregs and treats disease in several different mouse models (22, 34). In 2015, Spangler et al. solved the crystal structure of IL-2/JES6-1A12, showing that this IL-2 complex preferentially binds cells with the trimeric IL-2R (CD25, CD122, and common gamma chain) and not dimeric complexes (CD122 and common gamma chain), thus selecting for Tregs because of their constitutive CD25 expression (35).

Another approach to modulate IL-2 is to directly mutate IL-2 itself to change how it interacts with its receptor complex. Specifically, IL-2 “muteins” have alterations in the CD25-binding domain, thus decrease affinity for CD25, and enabling preferential binding to dimeric IL-2R complexes and activation of NK and CD8^+^ T cells (36–38). There is also much commercial interest in making IL-2 muteins with the opposite effect: IL-2 muteins that preferentially activate Tregs have led to a $400 million investment from Eli Lilly to Nektar Therapeutics and $300 million from Celgene to Delinia to develop this technology (39).

A final approach to modulate IL-2 signaling is to change IL-2R’s affinity for IL-2. Specifically, it is well established that single nucleotide polymorphisms in the *CD25* locus are associated with autoimmunity (40–43). Considering the power of CRISPR/Cas9 technology, in the future it could be possible to edit risk alleles of CD25 into protective alleles or otherwise engineer IL-2 signaling pathways to optimize therapeutic Treg function (44).

## Engineering Tregs with FOXP3

A hurdle in Treg therapy is generating sufficient numbers for clinical application (33). Since activated Tconv cells also express CD25 and FOXP3, and downregulate CD127, isolating Tregs on the basis of CD25 and CD127 alone introduces the risk of co-purifying and co-expanding non-Tregs. One way to overcome this limitation is to isolate naive CD45RA^+^CD25^hi^ cells from blood to enrich for a more homogeneous population (45, 46). However, this also significantly decreases the number of cells with which a culture can be started. Another potential solution to this problem is to isolate Tregs directly from the thymus for application as a third party cell therapy (47).

An additional approach is to find a way to engineer the desired Treg product. Indeed, the possibility of engineering Tregs *via* over-expression of FOXP3 has been considered since its discovery, with multiple studies showing that viral-mediated overexpression of FOXP3 in mouse or human T cells can induce suppressive function (48). Notably, in order to re-program human T cells into Tregs, FOXP3 has to be expressed at high and stable levels (49, 50); Treg suppressive capacity can be quickly reversed upon removal of FOXP3 (51).

Although FOXP3 is the master Treg transcription factor, evidence that its over-expression alone does not fully recapitulate the Treg gene signature led to the search for other co-factors and the discovery that co-expression of other transcription factors is important for full lineage specification (52). A consideration is whether studies which found that FOXP3 expression alone is not sufficient to induce a complete Treg gene signature considered the time that may be required for epigenetic re-programing to take place. Epigenetic modification and the consequent change in expression of other transcription factors is necessary to stabilize Treg phenotype and function (3). Since these epigenetic changes may require multiple rounds of cell division, re-programing Tconv cells into Tregs may not take place in short-term culture. The first application of FOXP3-engineered Treg therapy will likely happen as gene therapy for IPEX. CD4^+^ T cells from IPEX patients can be efficiently converted into functional and stable Tregs by FOXP3 gene transfer *in vitro* (53, 54). Testing these cells *in vivo* will rigorously determine if they have acquired sufficient Treg function to treat the severe autoimmunity in these patients.

## Engineering Antigen-Specificity

Antigen-specific Tregs have the benefit of being directed toward desired therapeutic antigens, thus increasing their potency up to 100-fold compared to polyclonal Tregs (55). Not only would fewer antigen-specific Tregs need to infused but they would also carry a lower risk of off-target suppression (55, 56). However, antigen-specific Tregs are extremely rare and must undergo significant *in vitro* expansion to achieve clinical doses. Despite this technical barrier, the testing of antigen-specific Tregs is already underway in the clinic in the context of organ transplantation (31).

Engineering antigen-specific Tregs by genomic modification to confer expression of desired transgenic T cell receptors (TCR) or by chimeric antigen receptors (CARs) represents an exciting approach to solve the challenge of the rarity of antigen-specific Tregs (57). Attempts to re-program the specificity of Tregs have been underway for several years. The first application in human Tregs involved gene transfer of a melanoma-specific, MHC Class I-restricted, TCR (58). These human TCR-transduced Tregs proliferated in response to antigen and suppressed antigen-specific Tconv cells *in vitro* and *in vivo*. Similarly, human Tregs transduced with a factor VIII (VIII)-specific TCR suppressed FVIII-specific Tconv cells and anti-FVIII antibody production from primed splenocytes (59). Human Tregs transduced with an islet antigen-specific TCR suppressed antigen-stimulated T cell responses. However, they were less efficient than Tregs expressing a viral antigen-specific TCR (60), possibly due to Treg-specific TCR affinity requirements (61). On the other hand, another study of human Tregs in which multiple class I-restricted TCRs recognizing the same peptide-MHC complex, but with affinities varying up to 3,500-fold, were tested, found TCR affinity had no effect on antigen-specific suppressive function (62). Thus, a consideration for future development of this approach is to find TCRs with an MHC restriction and specificity that would make them applicable in multiple patients, and which possess an optimal affinity for Tregs. TCRs which meet these requirements are most likely to be found in autoimmunity where there are well-known and relatively common MHC-peptide complexes that could be targeted.

## Chimeric Antigen Receptors

Another approach to engineer antigen-specific Tregs is to use a CAR technology, an idea borrowed from cancer immunotherapy. CARs were first described by Eshhar et al. in 1993 (63) and now being applied in humans for cancer immunotherapy (64–66). CARs give T cells the B-cell-like ability to bind to antigen in an MHC-independent manner. Additionally, the modular design of CARs allows for customization of specific regions, such as the signaling domains, to tailor the desired response from the engineered cell (67).

Over the last decade, a number of publications demonstrated the utility of CARs in Tregs (56). All reports used a standard second-generation design and included the CD28 co-stimulatory domain (Table 1) (68). Beginning with mouse models in 2008, Elinav et al. used Tregs from a mouse expressing a transgene for a hapten 2,4,6-trinitrophenol (TNP)-specific CAR (69). They found that transgenic TNP-specific CAR Tregs mediated antigen-specific suppression of effector T cells *in vitro* as well as *in vivo* resistance to colitis. The same group then demonstrated that the TNP-CAR could be introduced into mouse Tregs using retroviral-mediated gene transfer, giving these cells the ability to protect from disease *in vivo* in a dose-dependent manner (70). In a similar system, mouse CAR Tregs specific for a different model antigen, carcinoembryonic antigen (CEA), prevented disease in a model of colitis better than CAR Tregs specific for an irrelevant antigen. Importantly, these CEA-CAR Tregs homed to the location of the antigen (71).

**Table 1:** Summary of salient details from the current chimeric antigen receptor (CAR) regulatory T cells (Treg) publications.

Antigen and model disease	CAR structure	Species and expression system	Effects of CAR Treg therapy and points of significance	Reference
TNP Colitis	Hinge: CD28 TM: CD28 Co-stim: CD28 ITAMs: FcRγ	Mouse Transgene	– Protect from TNBS colitis – Bystander suppression of oxazolone-induced colitis – CD28 signaling required for CAR Treg function – *In vivo* imaging of Treg trafficking to site of inflammation	(69)
TNP Colitis	Hinge: CD28 TM: CD28 Co-stim: CD28 ITAMs: FcRγ	Mouse Retrovirus	– *Ex vivo* expansion through cognate antigen – Protect from TNBS colitis	(70)
Carcinoembryonic antigen (CEA) Sarcoma	Hinge: IgG Fc^a^ TM: CD28 Co-stim: CD28 ITAMs: CD3ζ	Human Retrovirus	– Suppression of CEA-specific antitumor response in humanized mouse model	(72)
Myelin oligodendrocyte glycoprotein Experimental autoimmune encephalomyelitis (EAE)	Hinge: IgG Fc^a^ TM: CD3ζ Co-stim:CD28^b^ ITAMs: CD3ζ	Mouse Lentivirus	– Dual expression system of FOXP3 and CAR – Reversal of EAE clinical symptoms, given at peak of disease	(73)
CEA Colitis	Hinge: IgG Fc^a^ TM: unknown Co-stim: CD28 ITAMs: CD3ζ	Mouse Retrovirus	– Protect from CEA-CAR T effector cell induced colitis – *In vivo* imaging of Treg trafficking to site of inflammation – Presence of CAR-specific antibodies correlated with disappearance of CAR Tregs	(71)
HLA-A2 Transplant rejection	Hinge: CD8α TM: CD28 Co-stim: CD28 ITAMs: CD3ζ	Human Lentivirus	– CAR-stimulated Tregs maintain stable phenotype – Suppression of alloantigen-specific T cells *in vitro* – Prevention of xenogeneic GVHD *in vivo*	(74)
HLA-A2 Transplant rejection	Hinge: CD28 TM: CD28 Co-stim: CD28 ITAMs: CD3ζ	Human Lentivirus	– Prevention of skin allograft rejection in humanized mouse model – Partial effect of CAR-lacking CD28 and CD3ζ intracellular signaling domains	(75)
HLA-A2 Transplant rejection	Hinge: CD8α TM: CD8 Co-stim: CD28 ITAMs: CD3ζ	Human Retrovirus	– Prevention of skin allograft rejection in humanized mouse models – CAR specificity tested against a panel of HLA-typed cells	(76)
Factor VIII Hemophilia A	Hinge: IgG Fc^a^ TM: CD28 Co-stim: CD28 ITAMs: CD3ζ	Human Retrovirus	– CAR directed against clinically-relevant soluble antigen – Suppression of recall antibody responses – Direct comparison between CAR and T cell receptor engineered Tregs	(77)

*A summary of the key features of the types of CARs that have been tested in Tregs. To date all CARs have utilized the CD28 co-stimulatory domain, but there are variations in the hinge and transmembrane (TM) regions employed. CARs containing Immune Tyrosine Activation Motifs (ITAMs) either from the FcRγ or CD3ζ proteins have been tested. All studies report superior effects of antigen-specific CAR Tregs compared to polyclonal or non-specific CAR Tregs*.

*^a^Hinge region presumed to be derived from IgG Fc*.

*^b^This CAR encoded CD3ζ amino-terminal to CD28*.

Apart from these studies in the context of IBD, there is currently only one other report of mouse CAR Tregs. Specifically, in 2012, Fransson et al. developed a CAR specific for myelin oligodendrocyte glycoprotein (MOG), the disease-causing agent for experimental autoimmune encephalomyelitis (EAE) (73). In this study, instead of isolating CD25^+^FOXP3^+^ Tregs, lentivirus was used to ectopically express FOXP3 and enforce a Treg phenotype. The resultant MOG-specific CAR Tregs suppressed responder T cell expansion *in vitro* and reversed symptoms of EAE. Overall, these publications provided important proof-of-concept data supporting the development of CAR Tregs for use in human cells.

Several publications have demonstrated the application of CAR technology to human Tregs. Three reports investigated the utility of expressing a CAR specific for HLA-A*02:01 (A2) to test whether CAR Tregs could be a new approach to control alloreactive T cells that cause rejection in HSCT and solid organ transplantation (74–76). The first publication showed that A2-CAR Tregs are activated and proliferate when stimulated through the CAR *via* coculture with A2-expressing cells (74). Additionally, A2-CAR Tregs prevented engraftment of A2^+^ PBMCs and development of xenogeneic GVHD in a humanized mouse model. Two other groups confirmed this approach, showing that A2-CAR Tregs suppress alloimmune responses better than polyclonal Tregs in humanized mouse models of A2^+^ skin xenografts (75, 76). A2 is an ideal antigen to target with CAR Tregs because it is broadly applicable in the transplant setting due to its high allelic frequency, meaning that a significant proportion of organ transplants could potentially benefit from this therapy (74). Moreover, HLAs in general are likely good targets for CAR Tregs since they are a membrane-bound protein specifically expressed on the transplanted tissues.

Yoon et al. reported the characterization of human CAR Tregs that target FVIII, the protein lacking in hemophilia which is immunogenic in patients receiving FVIII replacement therapy (77). Of specific interest from this study is the finding that a CAR specific for soluble antigens is suitable for use in Tregs, widening the possible antigen-targets that could be considered. This study also demonstrated that both T cell and antibody responses can be controlled by CAR Tregs. Also of note is that this study directly compared the effects of TCR versus CAR-engineered Tregs, finding that antibody recall responses were more effectively controlled by TCR-transgenic Tregs. More research is required to explore similarities and differences between TCR- and CAR-activated Tregs to better understand the affinity requirements and limitations of each approach (Table 2).

**Table 2:** Comparison of the benefits and limitations of engineering regulatory T cells (Tregs) to express a defined T cell receptor (TCR) versus chimeric antigen receptor (CAR), see also Harris and Krantz (57).

TCR	CAR
*Pros*: ✓ “Natural” protein; engineered cells should not be immunogenic ✓ Recall responses of TCR-transgenic Tregs may be more effective than CAR Tregs ✓ Designed to detect intracellular antigens ✓ Low affinity but high antigen sensitivity; fewer number of antigens required for TCR activation than CAR activation	*Pros*: ✓ MHC-independent antigen detection of soluble or cell-surface antigens ✓ Modular design enables more precise control over the type of antigen-stimulated response ✓ Hinge region provides flexibility, allowing CARs to bind antigen in a variety of orientations ✓ Higher antigen affinity than TCRs

*Cons*: – MHC-dependent peptide detection; each TCR complex has limited patient applicability – May require a large library of several TCR genes to adequately cover MHC/peptide complexes for one disease – Mispairing with endogenous TCRs could create new specificities and reduce efficacy	*Cons*: – “Unnatural” peptide sequence; construct may be immunogenic and limit ability to administer repeat doses – Ability to detect cell-surface antigens may be blocked by the presence of competing soluble antigen

## How Might Car Treg Behave in Humans?

Many of the fundamental properties of Tregs are similar to Tconv cells so it may be possible to predict some aspects of *in vivo* Treg behavior on the basis of findings from CAR Tconv cells used in the oncology field. However, Tregs also have many unique properties, such as their strict dependence on other cells for IL-2 and constitutive expression of inhibitory proteins such as CTLA-4 and TGF-β. Thus, there is a need for more detailed studies in animal models to fully appreciate the similarities and differences between the two cell types. For example, will CAR Tregs be able to persist long term even if their antigen is not available? Some research has shown that Tregs have different activation requirements than Tconv cells (62, 78), meaning that optimal proliferation and long-term persistence may require Treg-specific CAR design. Will CAR Tregs traffic to the necessary locations and mediate tolerance? CAR Tconv cells have been found to traffic to the lungs before moving to secondary lymphoid organs and disease sites, delaying their tumor-killing effect (79, 80). If there is similar phenomenon with Tregs then regional cell delivery may be preferred (79). Will CAR Tregs induce tolerance, and if yes, what molecular mechanisms will be necessary? CAR-activated Tregs upregulate CTLA-4, LAP, GARP, and CD39 (74), but it is unknown which pathway(s) are necessary for CAR Treg-mediated suppression. Further, what is the primary target of CAR Treg-mediated suppression? It is unknown whether CAR Tregs suppress immune cells at the site of inflammation, in secondary lymphoid organs, or both. Dissecting the mechanisms important to CAR Treg function may also provide clues as to their primary mode and location of immune suppression. Many of these questions are ideally suited for study in models of transplantation where similar questions with polyclonal or transgenic Tregs have been addressed (55).

## Next Steps: Where Will Engineered Treg Therapies Go from Here?

Many clinical trials with low-dose IL-2 therapies, expanded polyclonal and antigen-specific Tregs for use in autoimmune diseases, HSCT and solid organ transplantation are underway (18, 31, 33). While initial reports from these trials show that the treatments are well tolerated, the aggregate safety and efficacy data from each approach will greatly inform future studies. Notably, the possible long-term effects, and in particular the potential risk of cancer and infection, of these treatments will not be known for a significant period of time.

We predict that in the next ~5 years there will be a rapid transition from the rather crude current approaches with unmodified IL-2 and/or polyclonal Tregs to engineered approaches that enable precise control over the desired effect (81). It is likely that, as for low-dose IL-2 and polyclonal Treg therapy, transplantation will lead the way in testing these new engineered approaches. HSCT is a setting with a wealth of experience in using engineered T cells for cancer and it would be a natural transition to test engineered Tregs in this context. Moreover, in solid organ transplantation allogeneic HLA antigens represent an ideal target for antigen-specific Tregs because they are only expressed on the transplanted issue, minimizing the risk of off-target suppression (56). Additionally, since solid organ transplant donors and recipients are usually not HLA-matched, there is a large pool of patients that could benefit from this treatment. CAR targets for autoimmunity will be more difficult to identify because there are few truly organ and/or cell-specific antigens that would be suitable CAR targets. This challenge is similar to that faced in oncology, where off-target effects of CAR T cells can have devastating consequences (67, 82). The field of engineered Tregs will benefit greatly from the huge resources being invested into solving this problem in oncology (64–66), creating an ideal landscape to support the rapid development of this next generation of Treg therapies.

## Author Contributions

ND, JV-S, and ML reviewed the literature and wrote and revised the manuscript.

## Conflict of Interest Statement

The authors declare that the research was conducted in the absence of any commercial or financial relationships that could be construed as a potential conflict of interest.

## Abbreviations

CEA, carcinoembryonic antigen; EAE, experimental autoimmune encephalomyelitis; IPEX, immunodysregulation polyendocrinopathy enteropathy X-linked; HSCT, hematopoietic stem cell transplantation; GVHD, graft-versus-host disease; MOG, myelin oligodendrocyte glycoprotein; T1D, type 1 diabetes; Th, T helper; TNP, 2,4,6-trinitrophenol; Treg, regulatory T cell; Tconv, conventional T cell.
